# External beam radiation therapy in a centenarian with primary liver cancer: A case report

**DOI:** 10.1097/MD.0000000000022473

**Published:** 2020-11-20

**Authors:** Zhen Meng, Feifei Gao, Chang Liu, Shengcai Huang, Kai Hu, Rensheng Wang

**Affiliations:** a Department of Radiation Oncology, The First Affiliated Hospital of Guangxi Medical University; b Radiation Oncology Clinical Medical Research Center of Guangxi, Nanning, Guangxi, China; c Department of Radiology, The First Affiliated Hospital of Guangxi Medical University.

**Keywords:** case report, elderly patient, primary liver cancer, radiation therapy, super-elderly

## Abstract

**Rationale:**

Due to unprecedented global aging, the number of elderly and super-elderly patients with cancer is increasing. However, restricted by comorbidities or fragility, many elderly patients are considered ineligible to receive invasive therapies. A centenarian with primary liver cancer (PLC) was treated by external beam radiation therapy (EBRT). This rare case deserves our attention.

**Patient concerns:**

We present a rare case of a centenarian with PLC. The super-elderly male patient complained that 2 liver lesions were found by abdominal ultrasonography in June 2016.

**Diagnoses:**

The Segment 7 (S7) lesion and the Segment 5/8 (S5/8) lesion were clinically diagnosed as PLC successively.

**Interventions:**

The S7 lesion was considered PLC initially and treated by EBRT in October 2016. In the 1-year follow-up after EBRT, the S7 lesion was well controlled. Unfortunately, the S5/8 lesion had increased in size, was diagnosed as PLC and subsequently treated by CyberKnife in another hospital. However, local failure of the S5/8 lesion was suggested 15 months after CyberKnife. At the age of 102 years, the patient received re-irradiation for the S5/8 lesion.

**Outcomes:**

Three months after re-irradiation, des-γ-carboxy-prothrombin decreased to normal; no significant change in the S5/8 lesion was found in Magnetic Resonance Imaging. No severe acute or late toxicities were reported after each course of EBRT. Unfortunately, the patient died of respiratory failure caused by severe pneumonia in mid-March 2020.

**Conclusion:**

Advanced age is not a contraindication for elderly patients with cancer to receive radiotherapy and even re-irradiation.

## 1 Introduction

With an aggravated global aging problem and increased risk of cancer among the elderly, the group of cancer patients aged 65 and older is fast growing. While the highest age-specific incidence of primary liver cancer (PLC) among people aged 75 and older is in the USA, Canada, and the UK, the median age of PLC patients has increased by 10 years over the past 20 years in Japan.^[1,2]^ At present, surgical resection (SR) remains the most standard and reliable curative treatment for PLC; however, merely 0% to 14% of elderly patients are operable, due to the restriction of poor systemic condition or comorbidities.^[2]^ For super-elderly patients aged 85 years and above, nearly half (47%) have other serious diseases that greatly limit their access to invasive treatment.^[3,4]^ External beam radiation therapy (EBRT) is a non-invasive treatment option for them. However, there are scarce reports on the efficacy and safety of EBRT in elderly and super-elderly patients. We report a centenarian with the successful treatment of PLC by EBRT. The patient and his family gave the informed written consent for the report and publication.

## 2 Case report

**Figure 1:**
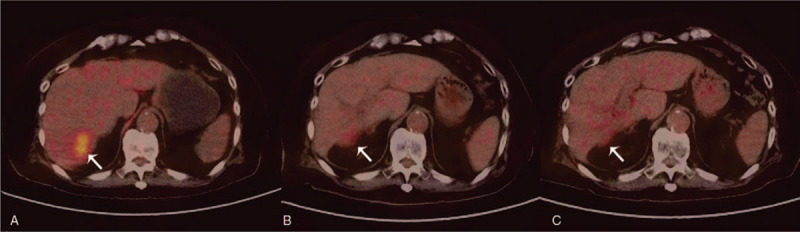
A: PET/CT showed an elevated 18F-FDG uptake (SUVmax 5.1) of the S7 lesion (June 2016). B: Reduction on size and decreased 18F-FDG uptake (SUVmax 2.7) was shown by PET/CT after EBRT 8 months (April 2017). C: Further reduction on size and normalized uptake were shown 1 year after EBRT (October 2017). 18F-FDG = fluorodeoxyglucose. SUV = standard uptake value.

In June 2016, a 99-year-old male was found 2 liver lesions by abdominal ultrasonography, 1 in Segment 7(4.3 × 4.2 cm) and the other 1 in Segment 5/8 (1.4 × 1.0 cm). Both lesions were suspected malignant with contrast-enhanced ultrasound. However, the multiphase abdominal computed tomography (CT) only showed the S7 lesion, following typical PLC enhancement imaging. The lesion in S7 also showed an elevated ^18^F- fluorodeoxyglucose uptake (standard uptake value max 5.1) on Positron Emission Tomography - CT (PET-CT, Fig. 1A), with no other lesions found. Liver biopsy revealed no malignant cells found by morphology or immunohistochemistry. The patient suffered delayed and local hemorrhage, and developed subcapsular and intrahepatic hematoma post-biopsy, preventing another biopsy to be admitted.

The case was with no obvious background of hepatitis B virus infection, family history of PLC or long-term history of alcoholism, and with negative α-fetoprotein (AFP) result and pathological results, however, aging and metabolic disease foundation, including diabetes, dyslipidemia, and hypothyroidism, were risk factors of PLC. Considered of the S7 lesion have the typical CT features of liver cancer: fast in and fast out, higher uptake on PET-CT, and the diameter reaching 3.5 cm, the patient was clinically diagnosed as PLC by multidisciplinary tumor panel. Because of his age and poor cardiopulmonary reserve, SR was not considered. Transarterial chemoembolization (TACE) was difficult because of obvious plaques of abdominal aortic atherosclerotic and celiac trunk malformations suggested by imaging. Considering the high risk of bleeding and that the size of the tumor exceeded 3 cm, radiofrequency ablation was not recommended. EBRT was recommended. However, the patient and their families refused EBRT and asked for the best supportive treatment and follow-up observation of changes of the lesions and the absorption of hematoma. Proprietary Chinese medicine and immunomodulatory therapy were given as adjuvant therapy.

In October 2016, it was found that the serum tumor marker AFP elevated to 41.19 ng/mL. At the same time, CT reexamination showed enlargement of the S7 lesion (6.4 cm × 5.7 cm), and no significant change in the S5/8 lesion size by ultrasound. With the dynamically increasing size on CT images, higher ^18^F- fluorodeoxyglucose uptake on PET-CT, and above normal AFP, the S7 lesion was given EBRT. A dose of 60 Gy in ten fractions was prescribed. The course of EBRT was completed smoothly without significant acute toxicity except for a complaint about transient anorexia.

**Figure 2:**
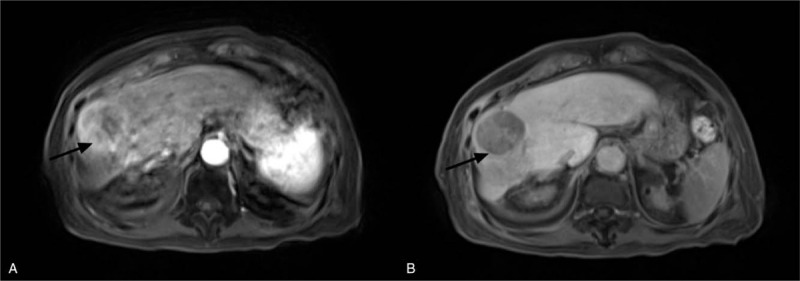
Fifteen months after the CyberKnife procedure (May 2019), contrast-enhanced MRI well-defined showed the local failure of S5/8. The lesion enlarged to 4.9 cm × 4.7 cm and showed arterial phase enhancement (A), followed by a washout in the venous phase (B).

**Figure 3:**
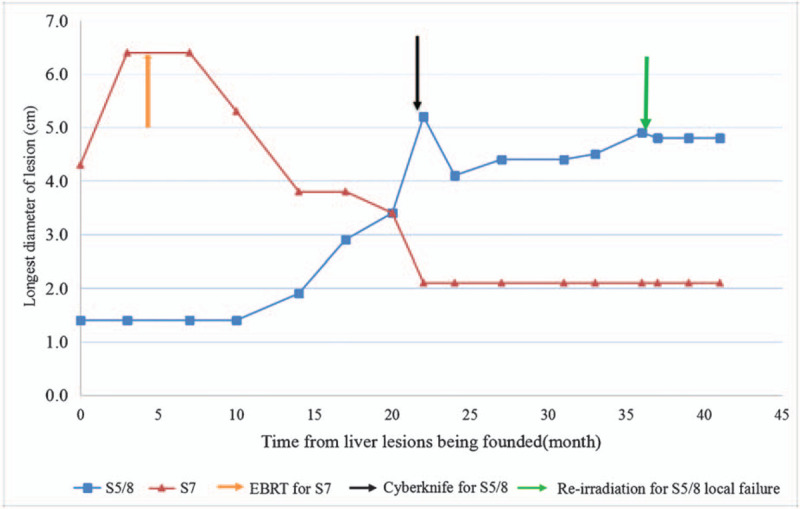
The change of the longest diameter of the lesion was measured by contrast-enhanced MRI or CT since June 2016; red solid line, longest diameter of S7 lesion (cm); blue solid line, longest diameter of S5/8 lesion (cm); orange arrow, EBRT on S7 lesion; black arrow, CyberKnife procedure for S5/8 lesion initially in another hospital; green arrow, re-irradiation on S5/8 lesion for the local failure.

The AFP level normalized after treatment. The S7 lesion showed continuous shrinkage with reduced arterial enhancement on CT and reduced uptake on PET-CT (Fig. 1B and C). After 1 year, CT suggested that the size of the S7 lesion was reduced by nearly 50%. Unfortunately, the S5/8 lesion enlarged and was treated by CyberKnife (42 Gy in 5 fractions) in another hospital in February 2018. Follow-up images showed that the S5/8 lesion had shrunk over time. However, in May 2019, 15 months after the CyberKnife procedure, local failure was suggested by magnetic resonance imaging (MRI), the S5/8 lesion had enlarged again (4.9 cm × 4.7 cm), the arterial phase enhancement was more pronounced (Fig. 2), and the des-γ-carboxy-prothrombin (DCP) value was elevated to 300 mAU/ml. Lenvatinib was initially administered orally for 1 week, but the patient discontinued the medication due to adverse effects of elevated blood pressure, thrombocytopenia, and fatigue. Although the patient had received 2 courses of radiotherapy, re-irradiation was performed for the S5/8 lesion (55 Gy in 10 fractions) as other treatments—such as SR, TACE, or targeted therapy—were not acceptable. No acute toxicities were found during the treatment period. At the patient's most recent follow-up, 3 months after the latest EBRT, liver function was normal and the DCP value had decreased to normal; MRI showed that the S5/8 lesion did not change significantly. Figure 3 showed the changes of the longest diameter of the 2 lesions in imaging. Regrettably, the patient died in mid-March 2020 from respiratory failure caused by severe pneumonia.

## 3 Discussion

EBRT is a non-invasive local treatment with fewer limitations of physical conditions. Whereas, the application of EBRT on PLC was limited in the past due to the possibility of radiation-induced liver disease (RILD), the advancement of image-guiding techniques, RT equipment, and computer engineering has highlighted EBRT as an important and precise treatment for liver cancer.^[5]^ In Korea, approximately 1 quarter of PLC patients receive EBRT.^[6]^ EBRT is much less affected by tumor location and tumor size than radiofrequency ablation, and has a higher local control (LC) rate than TACE.^[7]^ It can be applied to tumors ≥ 5 cm in size and achieve 74% of 1-year LC.^[8]^ Previous studies have confirmed the safety and feasibility of radiotherapy alone in elderly and super-elderly patients with cancer.^[9]^ A French multicenter analysis including 308 patients aged 90 and over suggested that radiation therapy with curative or palliative intent in nonagenarians was safe and effective.^[10]^ Therefore, advanced age is not a limitation of EBRT; even super-elderly PLC patients can be considered for EBRT treatment.

Charged particle therapy (CPT) using proton and heavier-ion beams can theoretically increase the dose of tumor and reduce the irradiated volume and dose of the normal liver and digestive tract. Therefore, CPT is expected to result in enhanced efficacy and lower toxicity of aged patients with PLC.^[11]^ Two small sample studies evaluated the outcome and the adverse events of patients aged ≥ 80 years (n = 21, 31 separately) with PLC who underwent proton beam therapy and carbon ion radiotherapy, respectively; both studies agreed with the efficacy and safety of CPT in PLC patients aged ≥ 80 years.^[11,12]^ However, the implementation of CPT is limited due to its economic burden and insufficient equipment at present.

In this case report, the treatment decision for the centenarian with PLC was made prudently. In terms of treatment safety, potential risk of worsening liver function and adverse effects were limitations of EBRT application. Whereas, EBRT was evaluated as a safe and effective option for Child-Pugh score A PLC patients; the patient had a good pretreatment liver function with child-pugh-A5. As we predicted, no liver function damage was found when the patient received either EBRT on the S7 lesion or re-irradiation on the S5/8 lesion. No other obvious acute or late radiation toxicities were found. Regarding EBRT efficiency, the S7 lesion was well controlled after EBRT. It was reported that a higher biologically effective dose was associated with improved LC; relatively low biologically effective dose may partially explain the local failure of the S5/8 lesion after CyberKnife.^[13]^ Three months after the re-irradiation of the S5/8 lesion, reexamination showed the DCP level had normalized and had a good performance status, with no change in the MRI image. However, typical imaging changes of the responding tumor, such as reduced enhancement and gradual size reduction, can appear over 6 months after treatment;^[14]^ in contrast, the serum tumor marker can be a more sensitive indicator.

Although it is a pity that the patient died of pneumonia about 9 months after the last radiotherapy, our case suggests that advanced age is not a contraindication for elderly patients with PLC to receive radiotherapy and even re-irradiation. EBRT may be a feasible alternative for those elderly patients who are inoperable or who refuse surgery.

## Acknowledgments

The authors thank the support by the Self-founding project of Gaungxi health Commission (NO. Z20170558), Guangxi Science and Technology Cooperation and Exchange Project (GKH 159905-2-11), and Guangxi Science and Technology Program Project (GK AD17129013). The authors would like to extend our heartfelt gratitude to the patient and his family, for understanding and willingness for publication.

## Author contributions

**Conceptualization**: Kai Hu, Rensheng Wang.

**Data curation**: Feifei Gao, Shengcai Huang.

**Visualization**: Chang Liu.

**Formal analysis**: Zhen Meng.

**Funding acquisition**: Kai Hu, Rensheng Wang.

**Writing – original draft**: Zhen Meng, Feifei Gao.

**Writing – review & editing**: Kai Hu, Rensheng Wang.
